# The economic burden of not meeting food recommendations in Canada: The cost of doing nothing

**DOI:** 10.1371/journal.pone.0196333

**Published:** 2018-04-27

**Authors:** Jessica R. L. Lieffers, John Paul Ekwaru, Arto Ohinmaa, Paul J. Veugelers

**Affiliations:** Population Health Intervention Research Unit, School of Public Health, University of Alberta, Edmonton, Alberta, Canada; McMaster University, CANADA

## Abstract

Few studies have estimated the economic burden of chronic diseases (e.g., type 2 diabetes, cardiovascular diseases, cancers) attributable to unhealthy eating. In this study, we estimated the economic burden of chronic disease attributable to not meeting Canadian food recommendations. We first obtained chronic disease risk estimates for intakes of both protective (1. vegetables; 2. fruit; 3. whole grains; 4. milk; 5. nuts and seeds) and harmful (6. processed meat; 7. red meat; 8. sugar-sweetened beverages) foods from the Global Burden of Disease Study, and food intakes from the 2004 Canadian Community Health Survey 24-hour dietary recalls (n = 33,932 respondents). We then calculated population attributable fractions (PAFs) for all relevant food-chronic disease combinations by age and sex groups. These PAFs were then mathematically combined for each disease for each age and sex group. We then estimated attributable costs by multiplying these combined PAFs with estimated 2014 annual direct health care (hospital, drug, physician) and indirect (human capital approach) costs for each disease. We found that not meeting recommendations for the eight foods was responsible for CAD$13.8 billion/year (direct health care: CAD$5.1 billion, indirect: CAD$8.7 billion). Nuts and seeds and whole grains were the top cost contributors rather than vegetables and fruit. Our findings suggest that unhealthy eating constitutes a tremendous economic burden to Canada that is similar in magnitude to the burden of smoking and larger than that of physical inactivity which were estimated using similar approaches. A status quo in promotion of healthy eating will allow this burden to continue. Interventions to reduce the health and economic burden of unhealthy eating in Canada may be more effective if they are broad in focus and include promotion of nuts and seeds and whole grains along with vegetables and fruit rather than have a narrow focus such as primarily on vegetables and fruit.

## Introduction

In Canada, chronic diseases (e.g., type 2 diabetes, cardiovascular diseases, cancers) were the leading causes of death in 2013 [1] and are responsible for billions of dollars in direct health care (i.e., hospitalization, drug, and physician costs) and indirect (i.e., lost productivity due to disability, morbidity, and premature mortality) costs each year based on the Economic Burden of Illness (EBIC) study [2]. With a population that is both aging and living longer, decreasing the burden of chronic diseases should be a priority to alleviate future tension on the health care system and improve overall productivity.

Several factors are known to cause chronic diseases including some that are non-modifiable (e.g., age, genetics). However, up to 80% of type 2 diabetes and cardiovascular diseases, and over 1/3 of cancers are caused by four modifiable behavioral risk factors: tobacco use, harmful alcohol use, physical inactivity, and unhealthy eating [3]. With various competing acute care demands and limited resources available for primary prevention, public health decision makers have difficult choices to make when allocating funds to achieve the best benefits in terms of avoiding health care costs and improving productivity.

Public health decision makers often consult cost of illness studies to understand the economic burden associated with different chronic disease risk factors [4]. To date, in Canada, estimates of the economic burden associated with different chronic disease risk factors have mostly focused on smoking [5–13], excess body weight [5–7,9,13–19], physical inactivity [5–7,9,13,14,20,21], and alcohol consumption [7,12,13,22]. For example, in 2013, the direct health care and indirect costs attributable to excess body weight, smoking, alcohol consumption, and physical inactivity were estimated to amount to CAD$23.5 billion, CAD$19.5 billion, CAD$10.6 billion, and CAD$9.3 billion, respectively [13]. Fewer studies have been conducted to estimate the economic burden associated with unhealthy eating despite many Canadians not meeting established food recommendations [23]. One possible reason there are few estimates is because this calculation is more complex due to both multiple risk factors and exposure levels.

One approach commonly used to estimate the economic burden associated with unhealthy eating is to quantify the impact of a single dietary factor [13,24–32]. For example, we recently estimated that the inadequate intake of vegetables and fruit costs Canadian society CAD$3.3 billion/year in direct health care and indirect costs [24]. Abdullah et al [32] found that if all adults in Canada met fibre recommendations, there would be potential savings in direct health care and indirect costs of CAD$1.3 billion/year for cardiovascular diseases and CAD$718.8 million/year for type 2 diabetes. Jones et al [31] reported that intake of sugar-sweetened beverages in Canada will cost CAD$33.7 billion over the next 25 years. However, because these studies focused on single dietary factors they underestimate the overall economic burden of chronic disease associated with unhealthy eating.

There have only been a few studies that estimated the overall economic burden of unhealthy eating. These studies have primarily been macroeconomic and ecologic in nature which do not consider food recommendations and specific dietary intakes. For example, in the United Kingdom, Scarborough et al [4] estimated that poor diets were responsible for £5.8 billion in National Health Services expenditures in 2006–07. Using a similar approach, Cancer Care Ontario and Public Health Ontario estimated that the direct health care costs associated with unhealthy eating in the Canadian province of Ontario were CAD$2.9 billion in 2011 [33]. Frazão et al [34] estimated that the economic burden of chronic diseases associated with unhealthy eating in the United States in 1995 was USD$70.9 billion in direct health care and indirect costs. Using an adapted approach [35], Health Canada estimated that the costs of unhealthy eating to the Canadian society in 1998 were $6.6 billion ($1.3 billion due to direct health care costs) [36].

The aim of this study was to estimate the economic burden of chronic disease attributable to unhealthy eating using both food recommendations and intakes from Canada. Unhealthy eating in this study was defined as not meeting established food recommendations.

## Materials and methods

This economic burden of not meeting food recommendations pertains to the mismatch between the amount of certain foods that is recommended and the amount that is actually consumed in Canada. It represents an estimate of costs that could potentially be avoided if all Canadians would fully comply with all food recommendations. We estimated the economic burden of not meeting food recommendations using a prevalence-based approach. This approach involved estimating the economic burden of chronic disease in a given period of time (i.e., one year) regardless of disease onset. Our estimations used a bottom up approach; this involved estimating the economic burden of different chronic diseases attributable to different foods for specific age and sex groups and then combining these estimates.

To obtain economic burden estimates, we first calculated population attributable fractions (PAF). Population attributable fractions use both the relative risk of disease due to a certain exposure and the distribution of the risk factor in a specific population (e.g., specific age and sex group) to estimate the fraction of disease cases that would not occur should the exposure be eliminated from the entire population (i.e., if everyone followed food recommendations). We then multiplied the PAF values by the overall economic costs associated with relevant chronic diseases to determine the estimated economic burden attributable to unhealthy eating.

We used multiple steps in our analyses which included: a) selection of foods for inclusion and extraction of dose-response relative risks; b) analysis of food consumption data; c) calculation of population attributable fractions; and d) estimation of attributable direct health care and indirect costs. These four steps are described in detail below.

Ethical approval for this study was provided by the University of Alberta Research Ethics Board (Pro00073196). The Canadian Community Health Survey data were accessed through Statistics Canada’s Research Data Centers (RDC) program.

### Food selection

We used foods in our analysis that were identified by the 2015 Global Burden of Disease Study (GBD) as having convincing or probable evidence against ≥1 chronic diseases [37]. The GBD summarized epidemiological evidence on various food-chronic disease combinations to identify those that either have convincing or probable evidence according to World Cancer Research Fund criteria for grading evidence [38]. We included the following eight foods in our analyses that had convincing or probable evidence against ≥1 chronic diseases: fruit not including juice, non-starchy vegetables, whole grains, nuts and seeds, fluid milk, red meat, processed meat, and sugar-sweetened beverages (SSB). We extracted dose-response relative risks for each of these foods for chronic disease morbidity from the 2015 GBD [37] with the exception of SSB which were extracted from the 2013 GBD (note: the relative risks for SSBs were estimated using a two-stage approach through body mass index) [39]. We used RRs for SSB from the 2013 GBD as the RR values in the 2015 GBD did not have the two-stage approach calculated RRs directly available. A list of all included food-chronic disease combinations is presented in Table 1. In addition to the food-chronic disease combinations in Table 1, the GBD had also identified additional combinations (fruit: nasopharyngeal cancer, other pharyngeal cancer; sugar-sweetened beverages: gallbladder cancer, hypertensive heart disease, cardiomyopathy, atrial fibrillation, peripheral vascular, endocarditis, and other cardiovascular). However, we did not consider their costs as information on the costs of these diseases was not available through the EBIC [2]. Because GBD age categories do not align exactly with EBIC age categories, we used the most conservative GBD relative risk estimates that applied to the relevant EBIC age category. A list of all relative risks used is presented in S1 Table.

**Table 1:** Food chronic disease combinations included in the analysis of the estimated economic burden of unhealthy eating in Canada.

Food	Chronic Disease	Food Recommendation	Serving Size	Canadian Nutrient File/Canada Food Guide subgroups and tiers included for analysis
**Fruit**	**Cancer**: Mouth, Larynx, Esophagus, Trachea, Lung, and Bronchus; **Cardiovascular Disease:** Ischemic Heart Disease, Ischemic Stroke, Hemorrhagic Stroke; **Diabetes**	Female and Male^†^: ≤14 years: 2 servings/day, 15+ years: 3 servings/day	80g	Vegetables and Fruit: Fruit other than juice (tiers 1–3; codes: 1121, 1122, 1123)
**Vegetables**	**Cardiovascular Disease:** Ischemic Heart Disease, Ischemic Stroke, Hemorrhagic Stroke	Female^†^: ≤14 years: 3 servings/day, 15+ years: 4 servings/day; Male^†^: ≤14 years: 3 servings/day, 15–54 years: 5 servings/day, 55+ years: 4 servings/day	80g	Vegetables and Fruit (note all corn and potatoes were removed from relevant categories): Vegetables, dark green (tiers 1–3; codes: 1211, 1212, 1213); Vegetables, deep yellow or orange (tiers 1–3; codes: 1221, 1222, 1223); Vegetables, other (tiers 1–3; codes: 1241, 1242, 1243)
**Whole Grains**	**Cardiovascular Disease:** Ischemic Heart Disease, Ischemic Stroke, Hemorrhagic Stroke; **Diabetes**	Female^††^: ≤14 years: 2.5 servings/day, 15+ years: 3 servings/day; Male^††^: ≤14 years: 2.5 servings/day, 15–54 years: 4 servings/day, 55+ years: 3.5 servings/day	35g	Grain Products: Whole grain (tiers 1–3; codes: 2101, 2102, 2103)
**Nuts and Seeds**	**Cardiovascular Disease:** Ischemic Heart Disease; **Diabetes**	30g (~1 Canada Food Guide serving)/day*	30g	Meat and Alternatives: Nuts and seeds (tiers 1–3; codes: 4601, 4602, 4603)
**Milk**	**Cancer:** Colon and Rectum	2 cups/day^††^	1 cup (~257.8g)	Milk and Alternatives (all soy beverages removed): Fluid milk and fortified soy-based beverages (tiers 1–3; codes: 3101, 3102, 3103)
**Red Meat**	**Cancer:** Colon and Rectum; **Diabetes**	No more than 3 X 85g servings/week (rounded this to ≤0.5 servings/day)**	75g	Meat and Alternatives (all offal and meat not meeting the International Agency for Research on Cancer criteria for red meat were excluded): Beef, game and organ meats (tiers 1–4; codes: 4101, 4102, 4103, 4104); Other meats (pork, veal, lamb) (tiers 1–4; codes: 4201, 4202, 4203, 4204)
**Processed Meat**	**Cancer:** Colon and Rectum; **Cardiovascular Disease:** Ischemic Heart Disease; **Diabetes**	Only for special occasions (assumed 0.05 servings/d servings/day)***	75g	Meat and Alternatives: Processed meats (tiers 1–4; codes: 4801, 4802, 4803, 4804)
**Sugar-Sweetened Beverages**	**Cancer:** Esophagus, Thyroid, Liver, Pancreas, Colon and Rectum, Breast (post-menopausal), Ovary, Uterus, Kidney, Leukemia; **Cardiovascular Disease:** Ischemic Heart Disease, Ischemic Stroke, Hemorrhagic Stroke; **Diabetes**; **Chronic Kidney Disease**	5g/day****	226.8g	Beverages sweetened with sugar with ≥50kcal/226.8g were included from the following subgroups: 5410 and 5420

^†^Based on Canada’s Food Guide and the 2015 GBD recommendations

^††^Based on Canada’s Food Guide recommendations

*Based on 2016 Canadian Cardiovascular Society Guidelines for the Management of Dyslipidemia for the Prevention of Cardiovascular Disease in the Adult

**Based on Canadian Cancer Society recommendations

***Based on Canadian Cancer Society and GBD 2015 recommendations

****Based on GBD 2015 recommendations

### Food consumption data analysis

Data on food consumption in the Canadian population were obtained from the Canadian Community Health Survey (CCHS) Cycle 2.2 (Nutrition) 2004, a cross-sectional national survey conducted by Statistics Canada and Health Canada from January 2004-January 2005 [40] which at the time this study was conducted was the most recent national survey of dietary intakes of Canadians available since the Nutrition Canada survey conducted in 1970–1972. Briefly, this survey encompasses a representative sample of individuals residing in private dwellings from the ten Canadian provinces. This survey represents about 98% of individuals living in these provinces and the response rate was 76.5%.

The 24-hour dietary recall was administered using an automated multi-pass method [41]. In total, 35,107 respondents completed a 24-hour dietary recall; of those respondents, 10,786 completed a second recall 3–10 days later. We eliminated the following records from all analyses: respondents <2 years, those marked as invalid, those where only breast milk was reported to be consumed, and records where no foods were recorded. In total, we included 44,325 24-hour dietary recall records that belonged to 33,932 respondents in our analyses. The Canadian Nutrient File (CNF 2001b) was used to determine the nutrient content of all reported foods.

Eating Well with Canada’s Food Guide (CFG) [42] was released in 2007 and outlines a dietary pattern for Canadians ≥2 years to help meet nutrient needs and prevent chronic diseases. Specific recommendations in terms of quantity and quality for four different food groups (Vegetables and Fruit, Grain Products, Milk and Alternatives, Meat and Alternatives) as well as other dietary factors (e.g., fats and oils, water) are outlined for nine different age and sex groups [42]. Because all foods included in our analyses except sugar-sweetened beverages are part of various CFG groups, we quantified the consumption of these foods using CFG servings.

To quantify the number of CFG servings from the 24-hour dietary recalls, we used the Canada Food Guide file which classifies all included foods in every 24-hour dietary recall according to Health Canada’s validated Canadian Nutrient File/Canada Food Guide (CNF/CFG) tool [43,44]. This tool classifies foods into the four CFG groups as well as into subgroups within those groups (e.g., Vegetables and Fruit subgroup examples: a) fruit other than juice, b) vegetables dark green). In addition, within those subgroups, foods are categorized into tiers based on quantities of fat, sodium, and sugar and adjustments based on other food guide guidance. Foods in Tiers 1–3 are considered to count towards CFG servings, and foods belonging in Tiers 4 are higher in fat, sugars, and/or sodium and are generally not counted towards CFG servings [43]. In addition, the CNF/CFG tool classifies some other foods (e.g., beverages).

A list of all food groups and subgroups included in our analyses are presented in Table 1. We included all foods belonging to each of the relevant subgroups with a few exceptions. For red meat, we only included foods from the a) beef, game and organ meats, and b) other meats (pork, veal, lamb) subgroups that met the International Agency for Research on Cancer red meat definition (i.e., meat from the muscle of mammals (e.g., pork, beef, veal, lamb)) [45]. We also excluded fortified soy beverages from the fluid milk and fortified soy-based beverages subgroup, and potatoes and corn from relevant vegetable subgroups [46]. For healthful foods (fruit, vegetables, whole grains, nuts and seeds, milk), we only included foods belonging to Tiers 1–3. For harmful foods (processed meat, red meat), we included foods belonging to Tiers 1–4. For beverages part of the CNF/CFG beverage subgroups, we considered those with ≥50kcal/226.8g to be a SSB [46]. We assumed standard CFG serving sizes for each included food, and that one serving of SSB was 226.8g (Table 1).

We assumed that meeting food recommendations in Canada set by government and other reputable organizations were associated with the lowest chronic disease risk. Canada’s Food Guide recommendations were used for whole grains and milk. Because CFG does not contain separate vegetable and fruit recommendations (only a recommendation for vegetables and fruit combined), we estimated these recommendations using the GBD 2015 Theoretical Minimum Risk Exposure (TMRE). We first added the TMRE values for vegetables and fruit (~800g/day) [37] and determined the fraction of this total for vegetables (~0.63) and fruit (~0.38). For each of the nine CFG age and sex groups, we then multiplied the total number of CFG servings for vegetables and fruit by these fractions, and rounded to the nearest serving to estimate separate vegetable and fruit serving recommendations. Because CFG age groups do not exactly align with age groups used in the EBIC to estimate economic costs, we made the following assumptions. First, for the EBIC age category encompassing individuals ≤14 years, we used the CFG recommendations for children 4–8 years as these recommendations were in the middle of this age category. For adults, we used the CFG recommendation that applied to the majority of the relevant EBIC age and sex group. For nuts and seeds, processed meat, and red meat, we used recommendations set by reputable Canadian organizations because CFG does not provide specific recommendations for these foods. We used a recommendation of 30g/day of nuts and seeds (~1 CFG serving of nuts and seeds) based on the 2016 Canadian Cardiovascular Society Guidelines for the Management of Dyslipidemia for the Prevention of Cardiovascular Disease in the Adult [47]. For processed meat and red meat, we used Canadian Cancer Society recommendations (processed meat: only for special occasions (assumed <0.05 servings/day based on the 2015 GBD TMRE); red meat: ≤3X85g cooked servings/week) [48]. We further assumed a recommendation of 5g/day of SSB based on the 2015 GBD TMRE. A summary of all recommendations we used is provided in Table 1.

We estimated usual intake distributions (distribution of long-term average daily intakes) for each age and sex group of interest using the National Cancer Institute (NCI) method [49] MIXTRAN and DISTRIB macros executed with SAS version 9.4 (SAS Inc. Cary, NC). The NCI method has the capability to estimate usual intake distributions of dietary factors consumed either non-episodically (i.e., by almost everyone), using a one-part amount model, and episodically (i.e., those not consumed daily by nearly everyone), using a two-part amount and probability model [49,50]. This method also allows for the adjustment of covariates. Similar to other studies using the NCI method to calculate usual intake distributions [51–53], we stratified the sample into three groups: children ≤14 years, women ≥15 years, and men ≥15 years. Also similar to previous studies [51–53], we used dummy variables for sex (child models only) and age groups (15–34 years; 35–54 years; 55–64 years; 65–74 years; 75+ years) (adult models only), weekend or weekday recall, and sequence (difference between first and second recall). We used the two-part model for all foods except vegetables (all models), and milk (child model only) where we used a one-part model. We used one-day intakes for nuts and seeds because so few respondents consumed this food.

Using the DISTRIB macro, we obtained information on the percentage of each EBIC age and sex group (males and females; ≤14 years, 15–34 years, 35–54 years, 55–64 years, 65–74 years, ≥75 years) consuming different levels of food intakes. For all foods except SSBs, we obtained information on proportions of the population consuming half CFG serving increments. For sugar-sweetened beverages, we obtained information on the proportion of the population consuming increments of 226.8g (one serving) with the exception of capturing information on half serving increments between one and two servings. For foods where the recommendation is near 0, we assumed that the proportion of the population consuming below the following cut-points was not associated with elevated risk: 0.25 servings/day of processed meat and 56.7g/day of SSB as accurate estimates below these values could not be obtained.

### Population attributable fraction calculations

We used the dose-response relative risks and food consumption data as inputs to calculate PAFs. The PAF uses both the relative risk of disease and the risk factor prevalence to estimate the fraction of disease that could theoretically be eliminated should the entire population follow healthy eating recommendations [6]. The standard PAF equation is as follows: P(RR-1)/[P(RR-1)+1] where P is the risk factor prevalence, and RR is the relative risk of disease. However, because food intakes are associated with different levels of disease risk depending on the degree to which recommendations are met, we used the method outlined by Krueger et al [6] to handle multiple risk exposure levels. The equation is as follows:

$$PAF=\frac{\sum_{i=1}^{n}P_{i}({RR}_{i}-1)}{1+\sum_{i=1}^{n}P_{i}({RR}_{i}-1)}$$

Where,

*P_i_* is the proportion of individuals in interval *i*,

*i* (interval) refers to the consumption of specific numbers of servings/day (e.g., <0.5, 0.5-<1, ≥1),

*RR* is the relative risk for each incremental increase in food consumption,

${RR}_{i}={RR}^{\left(X_{i}-L\right)}$ is the relative risk for interval *i* relative to the recommended number of servings,

*X_i_* is the mid value of interval *i*,

*L* is the recommended number of servings, and

*n* is the number of intervals above or below the recommended number of servings.

We calculated separate PAFs for each food for each chronic disease by sex for each of the following age groups: ≤14 years, 15–34 years, 35–54 years, 55–64 years, 65–74 years, and ≥75 years. The PAF values for each chronic disease were then combined for each sex and age group. Because some of the risk factors may be overlapping (i.e., several risk factors may be similar to one another and therefore redundant) and it is unclear what this overlap entails, it is important that we do not combine PAF values by adding them together as this would result in double counting. As suggested by Krueger et al [6], we used the following equation for this process: Combined PAF = 1-[(1-PAF_1_)(1-PAF_2_)(1-PAF_n_)]. This formula makes the assumption that the risk factors are independent (presence of one risk factor does not affect the probability of having another) and the joint effects of these risk factors are multiplicative (i.e. the presence of one factor does not affect the effect of another on a multiplicative scale). This approach has also been previously used to combine dietary risk factors for other outcomes (e.g., mortality) [37,54,55].

### Estimation of direct health care and indirect costs

The calculated PAF outcomes were used to estimate the direct health care and indirect costs attributable to unhealthy eating. Multiple different sources released in different years were used to estimate costs. First, the proportion of direct health care costs (hospital, physician, drug) associated with each relevant chronic disease by sex for different age groups (i.e., ≤14 years, 15–34 years, 35–54 years, 55–64 years, 65–74 years, and ≥75 years) were extracted from the 2008 EBIC [2] which contains the most recent estimates of this information. Because this resource does not provide separate costs for different types of diabetes, we assumed that type 2 diabetes was responsible for 0.96 of total diabetes costs [56]. Second, these proportions were then multiplied by the total hospital, physician, and drug costs reported by the 2014 National Health Expenditure Trends (i.e., 2014 costs in Canadian dollars) [57] to estimate more current direct health care costs for each relevant chronic disease for each sex and age group. The National Health Expenditure Trends resource contains information on the total hospital, physician, and drug costs for all diseases, age, and sex groups combined. Indirect costs were then estimated using the modified human capital approach as described by Krueger et al [6]. Briefly, using the EBIC 1998 [58] resource, a ratio of total direct health care to indirect costs (which includes costs associated with mortality, long-term disability, and short-term disability) was calculated for each included disease; these ratios are presented in Table 2. The indirect cost estimates in EBIC include discounted (5%) present value of lost productivity of all deaths during their estimated life expectancy, together with annual lost productivity by long term and short term disability. The used method utilizes age- and sex-specific rates of life expectancy, average annual earnings, workforce participation rates and values of unpaid work in Canadian provinces and territories [58]. The 2014 hospital, physician, and drug costs were then multiplied by this ratio to obtain indirect costs for each disease for each age and sex group. Third, the 2014 direct health care (hospital, physician, drug) and indirect costs were then multiplied by the relevant PAF to determine the costs attributable to unhealthy eating. Similar to Krueger et al [6,7], a disaggregation step was also applied to determine the economic burden attributable to individual foods.

**Table 2:** Ratio of indirect to direct health care costs from the 1998 economic burden of illness.

	Ratio of Indirect to Direct Costs
**Cancer**	4.78
**Cardiovascular Diseases**	1.71
**Endocrine and Related Diseases (for Diabetes)**	1.19
**Genitourinary Diseases (for Renal Disease)**	0.35

A sensitivity analysis was also conducted similar to Krueger et al [59]. This analysis was done by re-calculating all PAFs using the 95% confidence interval upper and lower boundary estimates for all included relative risks. We also re-calculated the PAFs with the assumption that all individuals consumed one half serving (or one serving for SSBs) closer to the recommendations to determine the potential annual cost difference.

## Results

Table 3 shows the percentage of the Canadian population ≥2 years by age group and sex who met each food recommendation. Food recommendations that were least often met were for nuts and seeds (range: 0.8%-4.3%), and for whole grains (range: 1.3%-9.1%). Compared to nuts and seeds and whole grains, more Canadians met recommendations for fruit (range: 5.2%-19.0%), vegetables (range: 5.8%-26.1%), and milk (range: 4.7%-37.8%). The percentage of the population consuming <56.7g of SSB/day ranged from 9.9% to 68.9%, the percentage consuming <0.25 servings/day of processed meat ranged from 22.4% to 84.0%, and the percentage consuming less than ≤0.5 servings of red meat/day ranged from 17.1% to 70.1%. On average, Canadians were more likely to meet recommendations for harmful foods than those of healthful foods. More detailed information about the percentage of the population consuming specific numbers of food servings by age group and sex is presented in S2 Table.

**Table 3:** Percentages of the 2004 Canadian population ≥2 years by age and sex meeting food recommendations.

	Canadian Population in 2014 (‘000)^†^	Healthful Foods (% consuming at or above recommendation)					Harmful Foods (% consuming at or below recommendation		
		Nuts and Seeds	Whole Grains	Fruit	Vegetables	Milk	Processed Meat^††^	Sugar-Sweetened Beverages*	Red Meat
**Females**									
≤14 years	2 780.6	1.1	4.8	19.0	15.9	27.6	49.3	15.8	70.1
15–34 years	4 703.0	1.5	1.6	5.8	5.8	9.7	67.0	24.6	59.2
35–54 years	4 989.7	3.1	1.6	8.7	13.4	6.0	70.6	48.7	44.2
55–64 years	2 378.0	4.3	2.4	8.9	15.7	4.7	78.4	57.7	42.0
65–74 years	1 629.2	1.9	1.5	9.0	13.1	5.5	73.2	61.7	48.0
75+ years	1 434.6	1.5	2.2	8.1	6.7	6.9	84.0	66.7	49.8

**Males**									
≤14 years	2 928.0	0.8	9.1	15.7	12.2	37.8	34.4	12.5	54.8
15–34 years	4 825.4	2.5	1.3	5.2	7.0	16.8	22.4	9.9	22.4
35–54 years	5 002.8	3.5	1.8	9.9	9.4	6.7	28.3	29.3	17.1
55–64 years	2 347.5	3.5	3.5	12.6	26.1	5.8	26.2	42.5	22.1
65–74 years	1 516.3	3.0	3.1	11.6	21.3	7.1	38.1	54.4	27.9
75+ years	1 005.1	2.8	2.8	9.4	17.4	11.8	51.2	68.9	36.3

^†^Statistics Canada. Table 2 Population by age group and sex, Canada, 2014. Available at: http://www.statcan.gc.ca/pub/89-503-x/2015001/article/14152/tbl/tbl2-eng.htm Accessed 22 Dec 2017.

^††^≤0.25 servings/day

*<56.7g/day

Combined PAF values for each disease by age and sex are presented in Table 4. Combined PAF values for males were generally higher compared to females. Combined PAF values for individuals ≥35 years for cardiovascular diseases and type 2 diabetes were higher (PAF range: 13.6%-74.6%) than those for various cancers and chronic kidney disease (PAF range: 0.03%-27.6%).

**Table 4:** Combined population attributable fractions for foods with established food recommendations presented by age, sex and chronic disease.

	Females							Males						Foods included in calculations
	≤14 years	15–34 years	35–54 years	55–64 years	65–74 years	75+ years		≤14 years	15–34 years	35–54 years	55–64 years	65–74 years	75+ years	
**Cancer**														
Mouth	2.6	6.1	5.4	5.1	5.0	5.0		3.0	6.6	5.7	5.2	5.1	5.4	**F**
Larynx	2.6	6.1	5.4	5.1	5.0	5.0		3.0	6.6	5.7	5.2	5.1	5.4	**F**
Thyroid	0.45	0.49	0.24	0.22	0.19	0.12		0.66	1.0	0.65	0.42	0.29	0.13	**SSB**
Trachea, Lung, and Bronchus	4.7	10.6	9.5	9.0	8.8	8.8		5.3	11.5	10.0	9.1	9.0	9.4	**F**
Esophagus	9.8	20.5	18.2	17.4	16.9	16.8		11.1	22.6	19.5	17.8	17.4	17.8	**F, SSB**
Liver	0.56	0.61	0.30	0.26	0.22	0.14		0.92	1.5	0.75	0.56	0.38	0.18	**SSB**
Pancreas	0.23	0.24	0.18	0.13	0.11	0.09		0.26	0.42	0.22	0.14	0.10	0.05	**SSB**
Colon and rectum	15.8	17.3	18.5	18.6	18.2	16.6		16.7	25.6	27.6	27.3	24.2	20.3	**M, RM, PM, SSB**
Kidney	0.90	0.97	0.53	0.44	0.37	0.26		0.79	1.3	0.65	0.49	0.34	0.16	**SSB**
Leukemia	0.45	0.49	0.24	0.22	0.15	0.12		0.26	0.42	0.22	0.21	0.10	0.05	**SSB**
Post-Menopausal Breast				0.13	0.11	0.09								**SSB**
Ovary	0.11	0.12	0.06	0.04	0.04	0.03								**SSB**
Uterus	1.6	1.7	0.88	0.79	0.67	0.46								**SSB**
**Cardiovascular Diseases**														
Ischemic Heart Disease	87.6	86.9	63.7	51.5	45.9	39.8		89.6	93.5	74.6	60.1	51.1	43.8	**F, V, WG, NS, PM, SSB**
Ischemic Stroke	77.8	90.5	61.0	44.7	32.3	13.6		78.4	95.0	70.1	47.6	34.2	14.8	**F, V, WG, SSB**
Hemorragic Stroke	64.5	80.6	55.5	44.2	35.1	16.1		65.4	87.5	64.4	47.0	37.2	17.5	**F, V, WG, SSB**
**Diabetes**	73.5	77.1	57.7	46.2	39.1	23.6		78.6	91.1	74.9	61.6	48.3	28.9	**F, WG, NS, RM, PM, SSB**
**Chronic Kidney Disease**	1.9	2.1	1.0	0.88	0.63	0.32		2.0	3.1	1.6	1.2	0.67	0.24	**SSB**

F = Fruit; V = Vegetables; M = Milk; WG = Whole Grains; NS = Nuts and Seeds; RM = Red Meat; PM = Processed Meat; SSB = Sugar-Sweetened Beverages

Not meeting recommendations for the eight foods that have established recommendations was estimated to be responsible for an economic burden of CAD$13.8 billion (CAD$5.1 billion in direct health care costs, CAD$8.7 billion in indirect costs). The estimated direct health care costs that were attributable to not meeting these food recommendations represents approximately 3.9% of all hospital, physician, and drug costs in Canada in 2014. About 1/3 of the estimated costs were attributable to females and 2/3 were attributable to males (females: CAD$1.7 billion/year direct health care costs; CAD$2.8 billion indirect costs; males: CAD$3.5 billion/year direct health care costs, CAD$5.8 billion/year indirect costs). Over 50% of these estimated direct health care and indirect costs were attributable to ischemic heart disease (CAD$2.9 billion direct health care costs; CAD$4.9 billion indirect costs); type 2 diabetes was also a substantial contributor (CAD$1.7 billion direct health care costs; CAD$2.1 billion in indirect costs). Fewer of these estimated costs were attributable to cancer, stroke, and chronic kidney disease (cancer: CAD$253.9 million direct health care costs, CAD$1.2 billion in indirect costs; stroke: CAD$281.1 million in direct health care costs, CAD$480.4 in indirect costs; chronic kidney disease: CAD$4.4 million in direct health care costs, CAD$1.6 million in indirect costs). Our sensitivity analysis that used the relative risk 95% confidence interval boundaries in the PAF calculations revealed 95% confidence interval for total estimated costs to be $6.9-$18.5 billion. For estimated direct health care costs this was $2.6-$6.8 billion, and for estimated indirect costs this was $4.3-$11.7 billion.

Table 5 shows the estimated economic burden for each of the eight foods that have established recommendations. The estimated economic burden of inadequate intakes of vegetables, fruit, whole grains, nuts and seeds, and milk exceeds the burden of excess intakes of red meat, processed meat, and sugar-sweetened beverages (CAD$10.6 billion vs. CAD$3.2 billion). Overall, >20% of estimated direct health care and indirect costs attributable to unhealthy eating were due to each of inadequate nuts and seeds and whole grains with both having $1.3 billion direct health care costs and $2.0 billion indirect costs. Excess processed meat and inadequate intakes of fruit were estimated to each be responsible for about 14–17% of direct health care and indirect costs attributable to unhealthy eating (fruit: $780.9 million direct heath care costs, $1.4 billion indirect costs; processed meat: $728.3 million direct health care costs, $1.2 billion indirect costs). Excess intakes of sugar-sweetened beverages and inadequate intakes of vegetables were estimated each to be responsible for 6–9% of these costs (sugar-sweetened beverages: $382.8 million direct health care costs, $480.4 million indirect costs; vegetables: $430.4 million direct health care costs, $735.7 million indirect costs). Milk and red meat were estimated to be responsible for lowest economic burden at ≤6% (red meat: $134.1 million direct health care costs, $263.4 million in indirect costs; milk: $112.3 million direct health care costs; $536.4 million indirect costs).

**Table 5:** Estimations of the economic burden in Canada in 2014 of the eight foods that have established recommendations.

	2014 estimated costs (‘000 $CAN)								
	Females			Males			Females and Males		
	Estimated Direct Health Costs	Estimated Indirect Costs		Estimated Direct Health Costs	Estimated Indirect Costs		Estimated Direct Health Costs	Estimated Indirect Costs	Estimated Total Direct Health and Indirect Costs
**Nuts and Seeds**	**468 741.4**	**721 012.5**		**814 751.2**	**1 295 273.4**		**1 283 492.6**	**2 016 285.9**	**3 299 778.5**
Ischemic Heart Disease	315 347.1	539 051.6		628 470.0	1 074 301.1		943 817.2	1 613 352.8	2 557 169.9
Diabetes	153 394.3	181 960.9		186 281.1	220 972.3		339 675.4	402 933.1	742 608.5
**Whole Grains**	**436 808.7**	**654 407.2**		**854 709.5**	**1 321 556.5**		**1 291 518.2**	**1 975 963.7**	**3 267 481.9**
Ischemic Heart Disease	210 694.1	360 158.7		521 006.6	890 604.1		731 700.8	1 250 762.8	1 982 463.6
Ischemic Stroke	28 340.0	48 444.1		41 400.7	70 770.0		69 740.7	119 214.2	188 954.9
Hemorrhagic Stroke	21 405.1	36 589.7		25 699.0	43 929.7		47 104.1	80 519.4	127 623.5
Diabetes	176 369.5	209 214.8		266 603.2	316 252.6		442 972.6	525 467.4	968 440.0
**Fruit**	**299 332.6**	**552 600.6**		**481 562.9**	**895 069.3**		**780 895.5**	**1 447 669.9**	**2 228 565.5**
Mouth Cancer	1 937.9	9 253.5		4 260.2	20 342.4		6 198.1	29 595.9	35 793.9
Laryngeal Cancer	532.8	2 544.2		2 291.9	10 943.8		2 824.7	13 487.9	16 312.6
Esophageal Cancer	3 266.0	15 595.1		10 873.7	51 922.0		14 139.7	67 517.1	81 656.8
Tracheal, Bronchial and Lung Cancer	23 506.1	112 242.1		26 060.1	124 437.3		49 566.2	236 679.4	286 245.7
Ischemic Heart Disease	121 538.8	207 757.4		257 726.6	440 555.7		379 265.5	648 313.1	1 027 578.6
Ischemic Stroke	31 323.4	53 543.9		38 630.4	66 034.4		69 953.7	119 578.3	189 532.0
Hemorrhagic Stroke	24 094.9	41 187.6		24 316.0	41 565.6		48 410.9	82 753.1	131 164.0
Diabetes	93 132.8	110 476.9		117 404.1	139 268.2		210 536.8	249 745.1	460 281.9
**Vegetables**	**146 173.6**	**249 867.9**		**284 230.4**	**485 860.9**		**430 404.0**	**735 728.7**	**1 166 132.7**
Ischemic Heart Disease	126 826.6	216 796.3		261 605.6	447 186.3		388 432.2	663 982.6	1 052 414.8
Ischemic Stroke	11 216.1	19 172.7		13 191.3	22 549.1		24 407.4	41 721.8	66 129.2
Hemorrhagic Stroke	8 130.9	13 898.8		9 433.5	16 125.5		17 564.3	30 024.3	47 588.7
**Milk**	**50 631.7**	**241 767.4**		**61 693.7**	**294 588.5**		**112 325.5**	**536 356.0**	**648 681.4**
Colon and Rectal Cancer	50 631.7	241 767.4		61 693.7	294 588.5		112 325.5	536 356.0	648 681.4
									
**Processed Meat**	**118 233.8**	**190 720.2**		**610 064.4**	**1 011 137.4**		**728 298.2**	**1 201 857.6**	**1 930 155.8**
Colon and Rectal Cancer	6 645.0	31 730.1		28 338.4	135 316.3		34 983.4	167 046.4	202 029.8
Ischemic Heart Disease	50 883.3	86 979.5		355 072.8	606 958.3		405 956.2	693 937.9	1 099 894.0
Diabetes	60 705.4	72 010.6		226 653.2	268 862.8		287 358.6	340 873.4	628 232.0
**Sugar-Sweetened Beverages**	**132 596.7**	**168 306.4**		**250 196.7**	**312 103.5**		**382 793.4**	**480 409.9**	**863 203.2**
Esophageal Cancer	78.4	374.5		345.7	1 650.5		424.1	2 025.0	2 449.1
Thyroid Cancer	126.4	603.6		84.9	405.5		211.3	1 009.1	1 220.4
Liver Cancer	16.6	79.2		107.8	514.9		124.4	594.1	718.5
Pancreatic Cancer	70.3	335.6		74.7	356.9		145.0	692.5	837.5
Colon and Rectal Cancer	266.4	1 271.9		1 153.5	5 507.8		1 419.8	6 779.7	8 199.5
Post-Menopausal Breast Cancer	538.8	2 572.7					538.8	2 572.7	3 111.5
Ovarian Cancer	40.7	194.2					40.7	194.2	234.9
Uterine Cancer	593.1	2 832.2					593.1	2 832.2	3 425.4
Kidney Cancer	446.8	2 133.4		258.0	1 231.8		704.8	3 365.2	4 070.0
Leukemia	336.9	1 608.5		289.6	1 383.1		626.5	2 991.6	3 618.1
Ischemic Heart Disease	5 157.1	8 815.5		15 240.9	26 052.6		20 398.0	34 868.2	55 266.1
Ischemic Stroke	620.4	1 060.5		1 057.7	1 808.1		1 678.2	2 868.6	4 546.8
Hemorrhagic Stroke	889.7	1 520.8		1 304.6	2 230.0		2 194.3	3 750.8	5 945.1
Diabetes	121 621.0	144 270.5		227 637.1	270 030.0		349 258.1	414 300.4	763 558.6
Chronic Kidney Disease	1 794.2	633.1		2 642.1	932.3		4 436.3	1 565.4	6 001.7
**Red Meat**	**27 146.3**	**54 516.7**		**106 994.3**	**208 839.9**		**134 140.7**	**263 356.6**	**397 497.2**
Colon and Rectal Cancer	6 217.9	29 690.8		22 826.7	108 997.7		29 044.6	138 688.5	167 733.1
Diabetes	20 928.4	24 825.9		84 167.7	99 842.2		105 096.0	124 668.1	229 764.1
**TOTAL ESTIMATED COSTS**	**1 679 664.9**	**2 833 198.9**		**3 464 203.1**	**5 824 429.4**		**5 143 867.9**	**8 657 628.3**	**13 801 496.2**

We also calculated the estimated cost savings if Canadians who do not meet recommendations for any of the eight foods improved their dietary choices by consuming half a serving closer to the recommendations for fruit, vegetables, milk, whole grains, nuts and seeds, red meat, and processed meat, and everyone who did not meet recommendations for sugar-sweetened beverages consumed one serving closer to the recommendations. In this scenario, the estimated economic burden would be $8.9 billion (direct health care: $3.3 billion; indirect: $5.5 billion), which is $4.9 billion (direct health care: $1.8 billion; indirect: $3.1 billion) less relative to the burden without this assumption. Because severe allergies to nuts are a concern in Canada [60], we repeated this calculation without any changes with respect to nuts and seeds. In this scenario, the estimated economic burden was estimated to be $10.8 billion (direct health care: $4.1 billion; indirect: $6.8 billion).

## Discussion

We estimated that CAD$13.8 billion in direct health care and indirect costs (which could be as low as CAD$6.9 billion and as high as CAD$18.5 billion) in 2014 were associated with not meeting Canadian food recommendations. Our analysis revealed that the largest contributors to this economic burden were inadequate intakes of nuts and seeds and whole grains. These estimates are insightful for public health professionals when considering the range of potential policy options and programs that aim to reduce the health and economic burden of chronic diseases.

Our estimate of CAD$13.8 billion for the economic burden of not meeting food recommendations suggests that investments in promotion of healthy eating have the potential of substantial savings in direct health care and indirect costs in Canada. Krueger et al [13] estimated that the economic burdens attributable to smoking, alcohol consumption, and physical inactivity in Canada were CAD$19.5 billion, CAD$10.6 billion, and CAD$9.3 billion, respectively, in 2013. Similar to our approach, theirs involved use of population attributable fractions and comparable methods to estimate costs. However, it is important to note differences between our estimates and those by Krueger et al [13]. Our focus was strictly on costs associated with the treatment and management of these chronic diseases. We had therefore only considered physician, hospital care and drug costs for these chronic diseases in our estimations. Krueger et al [13] took a broader perspective to costs to health care and therefore also considered costs for other health professionals (excluding dental), other health care expenditures, and health research. If we had considered the broader range of costs used by Krueger et al [13] our estimates would have been 30% to 50% higher. Adding 30% or 50% to our estimate of CAD$13.8 billion (increase to $17.9 billion or $20.7 billion) would put the economic burden of unhealthy eating at the same level than smoking and above physical inactivity estimated by Krueger et al [13]. The economic burden of unhealthy eating combined with that of other modifiable behavioral risk factors (i.e., smoking, alcohol consumption, physical inactivity) would be estimated to cost Canadian society in excess of $50 billion/year [13]. A status quo in promotion of these health behaviors will cause this enormous burden to continue. We refer to this as the ‘costs of doing nothing’ but recommend action.

Several previous studies conducted both in Canada [13,24,31,32] and internationally [4,25,26,28–30,34] have provided estimates for the economic burden of unhealthy eating. These studies have used various approaches and methodologies (e.g., top-down vs. bottom-up approaches; inclusion of varied types and numbers of dietary factors and types of costs) and have been conducted in different countries (each with different health care systems, chronic disease prevalence rates, and population distributions), which makes comparisons across studies difficult. Our study is unique as we used a bottom-up approach that considered all foods for which established recommendations exit, considered all chronic diseases for which scientific evidence exit, and considered age and sex sub-groups to add precision to the estimates. The only other estimate for Canada that was not limited to a single food item had followed a top down approach, i.e. did not consider specific food recommendations and dietary intakes. This study estimated the economic burden 20 years ago to be $6.6 billion ($1.3 billion in direct health care costs) [36].

The estimated economic burden associated with the consumption of the eight different foods are the result of a combination of their relative risk of disease, and the percentage of the population not meeting recommendations. These estimates help to draw attention to dietary intakes that are associated with the greatest burden of disease (e.g., nuts and seeds; whole grains). These estimates also illustrate that relatively small positive changes in dietary choices at the population level can lead to substantial cost savings. However, our estimates do not provide decision makers with actionable solutions on how to decrease the economic burden of disease. Although the estimated costs of inadequate nuts and seeds and whole grains exceeds that of vegetables and fruit, decision makers will also have to consider the possibility that it may be more difficult to increase intakes of nuts and seeds and whole grains because fewer Canadians meet those intakes compared to vegetables and fruit. The estimates from this study are useful to help guide the direction of future intervention and simulation studies examining the cost-effectiveness and cost-benefits of various programs and policy options (including costs to the consumer of eating more and specific types of healthy foods) to improve dietary intake. These studies, once completed, will provide guidance on how to best move forward with programs and policies to best decrease the burden of chronic diseases associated with unhealthy eating.

This study has several strengths. Dietary intakes were assessed using 24-hour dietary recalls of a representative sample of 33,932 Canadians. As well, only foods that cause chronic disease as identified by the GBD were included. A limitation is that our calculated value is likely an underestimate as the costs of certain chronic diseases and also costs associated with nutrients (e.g., fibre, fat, sodium) were not included. In addition, dietary assessment is a difficult process and underreporting unhealthy foods and over reporting of healthy foods is a common phenomenon. However, even with this limitation, the estimated costs associated with not meeting food recommendations were substantial. We also assumed that the proportion of costs associated with different diseases and ratio of direct health care to indirect costs remained consistent over time which may not be the case; however, these assumptions have been used in other similar Canadian studies [5,7,13,24]. We also did not examine regional differences in costs which have been previously reported to vary for other behavioral chronic disease risk factors in Canada (e.g., smoking, excess body weight, and physical inactivity [5]). In addition, these results do not show the estimated lifetime costs of not meeting food-based recommendations in Canada which is a limitation in all prevalence-based cost-of-illness studies. Using an incidence-based approach which is more complex would allow the lifetime costs to be estimated. A further limitation is that the dietary intake data used for this study was collected in 2004; however, this was the most recent data that is available with information on dietary intakes from a representative sample of Canadians at the time this study was conducted. We encourage researchers to use national nutrition datasets in their jurisdictions to estimate the economic burden of not meeting their nation’s food recommendations using a similar approach.

## Conclusions

The economic burden of not meeting food recommendations in Canada was estimated to be $13.8 billion in 2014. This estimate allows the impact of our food choices to be compared to estimates associated with other behavioral lifestyle choices (e.g., smoking, physical inactivity) determined using similar approaches. Although making dietary changes is a complex process which requires strong support and major investments from government and society, policies and programs which have the ability to make small positive changes at the population level have the potential to reduce the currently tremendous economic burden.

## Supporting information

S1 Table Relative risks used in analyses. (DOCX)

S2 Table Percentages of the 2004 Canadian population ≥2 years by age and sex, and by level of food intake. (DOCX)
