# Supplementary material for: The economic burden of not meeting food recommendations in Canada: The cost of doing nothing
Source: PLoS One. 2018 Apr 27;13(4):e0196333. doi: 10.1371/journal.pone.0196333 (PMC5922758; doi:10.1371/journal.pone.0196333)
Supplement: S1 Table — (DOCX) [file pone.0196333.s001.docx]

**S1 Table: Relative risks used in analyses**

**Females**

|  | **≤14 years** | **15-34 years** | **35-54 years** | **55-64 years** | **65-74 years** | **75+ years** |
| --- | --- | --- | --- | --- | --- | --- |
| **Fruit (per 100g)** |  |  |  |  |  |  |
| Mouth Cancer | 0.96 (0.92, 1.01) | 0.96 (0.92, 1.01) | 0.96 (0.92, 1.01) | 0.96 (0.92, 1.01) | 0.96 (0.92, 1.01) | 0.96 (0.92, 1.01) |
| Laryngeal Cancer | 0.96 (0.91, 1.01) | 0.96 (0.91, 1.01) | 0.96 (0.91, 1.01) | 0.96 (0.91, 1.01) | 0.96 (0.91, 1.01) | 0.96 (0.91, 1.01) |
| Esophageal Cancer | 0.87 (0.78, 0.97) | 0.87 (0.78, 0.97) | 0.87 (0.78, 0.97) | 0.87 (0.78, 0.97) | 0.87 (0.78, 0.97) | 0.87 (0.78, 0.97) |
| Tracheal, Bronchial and Lung Cancer | 0.93 (0.89, 0.97) | 0.93 (0.89, 0.97) | 0.93 (0.89, 0.97) | 0.93 (0.89, 0.97) | 0.93 (0.89, 0.97) | 0.93 (0.89, 0.97) |
| Ischemic Heart Disease (IHD) | 0.80 (0.69, 0.93) | 0.83 (0.73, 0.94) | 0.90 (0.84, 0.96) | 0.92 (0.87, 0.97) | 0.93 (0.89, 0.98) | 0.95 (0.91, 0.98) |
| Ischemic Stroke | 0.49 (0.35, 0.68) | 0.55 (0.41, 0.72) | 0.75 (0.65, 0.85) | 0.82 (0.74, 0.90) | 0.87 (0.82, 0.93) | 0.95 (0.92, 0.97) |
| Hemorrhagic Stroke | 0.59 (0.46, 0.76) | 0.63 (0.51, 0.79) | 0.77 (0.67, 0.87) | 0.82 (0.74, 0.90) | 0.86 (0.80, 0.92) | 0.94 (0.91, 0.97) |
| Diabetes | 0.89 (0.81, 0.97) | 0.89 (0.81, 0.97) | 0.91 (0.85, 0.98) | 0.93 (0.87, 0.98) | 0.94 (0.90, 0.99) | 0.97 (0.94, 0.99) |
| **Vegetables (per 100g)** |  |  |  |  |  |  |
| IHD | 0.80 (0.69, 0.92) | 0.83 (0.73, 0.93) | 0.90 (0.84, 0.96) | 0.92 (0.87, 0.97) | 0.93 (0.89, 0.97) | 0.95 (0.91, 0.98) |
| Ischemic Stroke | 0.80 (0.68, 0.95) | 0.83 (0.72, 0.96) | 0.91 (0.85, 0.98) | 0.94 (0.90, 0.99) | 0.96 (0.93, 0.99) | 0.98 (0.97, 1.00) |
| Hemorrhagic Stroke | 0.85 (0.75, 0.96) | 0.87 (0.78, 0.96) | 0.92 (0.87, 0.98) | 0.94 (0.90, 0.98) | 0.95 (0.92, 0.99) | 0.98 (0.97, 1.00) |
| **Whole Grains (per 50g)** | | | | | | |
| IHD | 0.68 (0.58, 0.79) | 0.72 (0.63, 0.82) | 0.84 (0.78, 0.90) | 0.88 (0.83, 0.92) | 0.90 (0.86, 0.94) | 0.91 (0.88, 0.94) |
| Ischemic Stroke | 0.48 (0.40, 0.60) | 0.54 (0.45, 0.65) | 0.77 (0.71, 0.83) | 0.84 (0.80, 0.88) | 0.90 (0.87, 0.93) | 0.96 (0.95, 0.97) |
| Hemorrhagic Stroke | 0.63 (0.55, 0.71) | 0.67 (0.60, 0.75) | 0.81 (0.76. 0.86) | 0.85 (0.81, 0.89) | 0.89 (0.86, 0.92) | 0.95 (0.94, 0.97) |
| Diabetes | 0.81 (0.74, 0.89) | 0.82 (0.75, 0.89) | 0.85 (0.80, 0.91) | 0.88 (0.83, 0.93) | 0.90 (0.86, 0.94) | 0.94 (0.91, 0.97) |
| **Nuts and Seeds (per 4.05g)** | | | | | | |
| IHD | 0.85 (0.76, 0.95) | 0.87 (0.79, 0.96) | 0.93 (0.88, 0.98) | 0.95 (0.91, 0.98) | 0.96 (0.93, 0.99) | 0.96 (0.94, 0.99) |
| Diabetes | 0.95 (0.93, 0.98) | 0.95 (0.93, 0.98) | 0.96 (0.95, 0.98) | 0.97 (0.96, 0.98) | 0.98 (0.96, 0.99) | 0.99 (0.98, 0.99) |
| **Milk (per 226.8g)** |  |  |  |  |  |  |
| Colorectal Cancer | 0.90 (0.83, 0.96) | 0.90 (0.83, 0.96) | 0.90 (0.83, 0.96) | 0.90 (0.83, 0.96) | 0.90 (0.83, 0.96) | 0.90 (0.83, 0.96) |
|  |  |  |  |  |  |  |
| **Red Meat (per 100g)** |  |  |  |  |  |  |
| Colorectal Cancer | 1.17 (1.03, 1.31) | 1.17 (1.03, 1.31) | 1.17 (1.03, 1.31) | 1.17 (1.03, 1.31) | 1.17 (1.03, 1.31) | 1.17 (1.03, 1.31) |
| Diabetes | 1.32 (1.04, 1.60) | 1.31 (1.04, 1.59) | 1.24 (1.03, 1.43) | 1.19 (1.02, 1.35) | 1.15 (1.02, 1.27) | 1.09 (1.01, 1.15) |
| **Processed Meat (per 50g)** | | | | | | |
| Colorectal Cancer | 1.18 (1.09, 1.27) | 1.18 (1.09, 1.27) | 1.18 (1.09, 1.27) | 1.18 (1.09, 1.27) | 1.18 (1.09, 1.27) | 1.18 (1.09, 1.27) |
| IHD | 2.57 (1.05, 4.70) | 2.12 (1.04, 3.50) | 1.52 (1.02, 2.05) | 1.42 (1.02, 1.83) | 1.35 (1.02, 1.69) | 1.25 (1.01, 1.48) |
| Diabetes | 1.94 (1.39, 2.56) | 1.91 (1.38, 2.51) | 1.65 (1.28, 2.05) | 1.51 (1.23, 1.80) | 1.39 (1.18, 1.61) | 1.22 (1.10, 1.33) |
| **Sugar Sweetened Beverage (per 226.8g)** | | | | | | |
| Esophageal Cancer | 1.008 (1, 1.018) | 1.008 (1, 1.018) | 1.009 (1, 1.02) | 1.011 (1, 1.023) | 1.011 (1, 1.023) | 1.01 (1, 1.021) |
| Thyroid Cancer | 1.004 (1.002, 1.005) | 1.004 (1.002, 1.005) | 1.004 (1.003, 1.006) | 1.005 (1.003, 1.007) | 1.005 (1.003, 1.007) | 1.004 (1.003, 1.006) |
| Liver Cancer | 1.005 (1.001, 1.009) | 1.005 (1.001, 1.009) | 1.005 (1.001, 1.01) | 1.006 (1.001, 1.011) | 1.006 (1.001, 1.011) | 1.005 (1.001, 1.01) |
| Pancreatic Cancer | 1.002 (1.001, 1.004) | 1.002 (1.001, 1.004) | 1.003 (1.001, 1.005) | 1.003 (1.001, 1.006) | 1.003 (1.001, 1.005) | 1.003 (1.001, 1.005) |
| Colorectal Cancer | 1.002 (1.001, 1.003) | 1.002 (1.001, 1.003) | 1.002 (1.001, 1.003) | 1.002 (1.001, 1.003) | 1.002 (1.001, 1.003) | 1.002 (1.001, 1.003) |
| Breast Cancer |  |  |  | 1.003 (1.001, 1.005) | 1.003 (1.001, 1.005) | 1.003 (1.001, 1.005) |
| Ovarian Cancer | 1.001 (1, 1.002) | 1.001 (1, 1.002) | 1.001 (1, 1.003) | 1.001 (1, 1.003) | 1.001 (1, 1.003) | 1.001 (1, 1.003) |
| Uterine Cancer | 1.014 (1.009, 1.019) | 1.014 (1.009, 1.019) | 1.015 (1.011, 1.022) | 1.018 (1.012, 1.026) | 1.018 (1.012, 1.025) | 1.016 (1.011, 1.023) |
| Kidney Cancer | 1.008 (1.005, 1.011) | 1.008 (1.005, 1.011) | 1.009 (1.006, 1.013) | 1.01 (1.007, 1.015) | 1.01 (1.007, 1.015) | 1.009 (1.006, 1.014) |
| Leukemia | 1.004 (1.002, 1.006) | 1.004 (1.002, 1.006) | 1.004 (1.002, 1.006) | 1.005 (1.002, 1.008) | 1.004 (1.002, 1.008) | 1.004 (1.002, 1.007) |
| IHD | 1.023 (1.006, 1.043) | 1.021 (1.007, 1.039) | 1.015 (1.01, 1.022) | 1.013 (1.008, 1.019) | 1.01 (1.006, 1.015) | 1.005 (1.003, 1.009) |
| Ischemic Stroke | 1.025 (1.009, 1.045) | 1.024 (1.011, 1.042) | 1.018 (1.011, 1.027) | 1.014 (1.009, 1.021) | 1.01 (1.006, 1.014) | 1.002 (1, 1.005) |
| Hemorrhagic Stroke | 1.032 (1.015, 1.053) | 1.032 (1.015, 1.053) | 1.025 (1.015, 1.038) | 1.019 (1.012, 1.03) | 1.013, 1.007, 1.019) | 1.002 (1, 1.007) |
| Diabetes | 1.46 (1.22, 1.75) | 1.43 (1.18, 1.70) | 1.30 (1.14, 1.48) | 1.24 (1.12, 1.38) | 1.19 (1.09, 1.29) | 1.12 (1.10, 1.15) |
| Chronic Kidney Disease | 1.017 (1.001, 1.036) | 1.017 (1.001, 1.036) | 1.017 (1.001, 1.036) | 1.020 (1.001, 1.041) | 1.017 (1.002, 1.034) | 1.011 (1, 1.031) |

**Note:** All dose-response relative risks were extracted from the 2015 GBD with the exception of sugar sweetened beverages which were extracted from the 2013 GBD **Males**

|  | **≤14 years** | **15-34 years** | **35-54 years** | **55-64 years** | **65-74 years** | **75+ years** |
| --- | --- | --- | --- | --- | --- | --- |
| **Fruit (per 100g)** |  |  |  |  |  |  |
| Mouth Cancer | 0.96 (0.92, 1.01) | 0.96 (0.92, 1.01) | 0.96 (0.92, 1.01) | 0.96 (0.92, 1.01) | 0.96 (0.92, 1.01) | 0.96 (0.92, 1.01) |
| Laryngeal Cancer | 0.96 (0.91, 1.01) | 0.96 (0.91, 1.01) | 0.96 (0.91, 1.01) | 0.96 (0.91, 1.01) | 0.96 (0.91, 1.01) | 0.96 (0.91, 1.01) |
| Esophageal Cancer | 0.87 (0.78, 0.97) | 0.87 (0.78, 0.97) | 0.87 (0.78, 0.97) | 0.87 (0.78, 0.97) | 0.87 (0.78, 0.97) | 0.87 (0.78, 0.97) |
| Tracheal, Bronchial and Lung Cancer | 0.93 (0.89, 0.97) | 0.93 (0.89, 0.97) | 0.93 (0.89, 0.97) | 0.93 (0.89, 0.97) | 0.93 (0.89, 0.97) | 0.93 (0.89, 0.97) |
| Ischemic Heart Disease (IHD) | 0.80 (0.69, 0.93) | 0.83 (0.73, 0.94) | 0.90 (0.84, 0.96) | 0.92 (0.87, 0.97) | 0.93 (0.89, 0.98) | 0.95 (0.91, 0.98) |
| Ischemic Stroke | 0.49 (0.35, 0.68) | 0.55 (0.41, 0.72) | 0.75 (0.65, 0.85) | 0.82 (0.74, 0.90) | 0.87 (0.82, 0.93) | 0.95 (0.92, 0.97) |
| Hemorrhagic Stroke | 0.59 (0.46, 0.76) | 0.63 (0.51, 0.79) | 0.77 (0.67, 0.87) | 0.82 (0.74, 0.90) | 0.86 (0.80, 0.92) | 0.94 (0.91, 0.97) |
| Diabetes | 0.89 (0.81, 0.97) | 0.89 (0.81, 0.97) | 0.91 (0.85, 0.98) | 0.93 (0.87, 0.98) | 0.94 (0.90, 0.99) | 0.97 (0.94, 0.99) |
| **Vegetables (per 100g)** |  |  |  |  |  |  |
| IHD | 0.80 (0.69, 0.92) | 0.83 (0.73, 0.93) | 0.90 (0.84, 0.96) | 0.92 (0.87, 0.97) | 0.93 (0.89, 0.97) | 0.95 (0.91, 0.98) |
| Ischemic Stroke | 0.80 (0.68, 0.95) | 0.83 (0.72, 0.96) | 0.91 (0.85, 0.98) | 0.94 (0.90, 0.99) | 0.96 (0.93, 0.99) | 0.98 (0.97, 1.00) |
| Hemorrhagic Stroke | 0.85 (0.75, 0.96) | 0.87 (0.78, 0.96) | 0.92 (0.87, 0.98) | 0.94 (0.90, 0.98) | 0.95 (0.92, 0.99) | 0.98 (0.97, 1.00) |
| **Whole Grains (per 50g)** | | | | | | |
| IHD | 0.68 (0.58, 0.79) | 0.72 (0.63, 0.82) | 0.84 (0.78, 0.90) | 0.88 (0.83, 0.92) | 0.90 (0.86, 0.94) | 0.91 (0.88, 0.94) |
| Ischemic Stroke | 0.48 (0.40, 0.60) | 0.54 (0.45, 0.65) | 0.77 (0.71, 0.83) | 0.84 (0.80, 0.88) | 0.90 (0.87, 0.93) | 0.96 (0.95, 0.97) |
| Hemorrhagic Stroke | 0.63 (0.55, 0.71) | 0.67 (0.60, 0.75) | 0.81 (0.76. 0.86) | 0.85 (0.81, 0.89) | 0.89 (0.86, 0.92) | 0.95 (0.94, 0.97) |
| Diabetes | 0.81 (0.74, 0.89) | 0.82 (0.75, 0.89) | 0.85 (0.80, 0.91) | 0.88 (0.83, 0.93) | 0.90 (0.86, 0.94) | 0.94 (0.91, 0.97) |
| **Nuts and Seeds (per 4.05g)** | | | | | | |
| IHD | 0.85 (0.76, 0.95) | 0.87 (0.79, 0.96) | 0.93 (0.88, 0.98) | 0.95 (0.91, 0.98) | 0.96 (0.93, 0.99) | 0.96 (0.94, 0.99) |
| Diabetes | 0.95 (0.93, 0.98) | 0.95 (0.93, 0.98) | 0.96 (0.95, 0.98) | 0.97 (0.96, 0.98) | 0.98 (0.96, 0.99) | 0.99 (0.98, 0.99) |
| **Milk (per 226.8g)** |  |  |  |  |  |  |
| Colorectal Cancer | 0.90 (0.83, 0.96) | 0.90 (0.83, 0.96) | 0.90 (0.83, 0.96) | 0.90 (0.83, 0.96) | 0.90 (0.83, 0.96) | 0.90 (0.83, 0.96) |
|  |  |  |  |  |  |  |
| **Red Meat (per 100g)** |  |  |  |  |  |  |
| Colorectal Cancer | 1.17 (1.03, 1.31) | 1.17 (1.03, 1.31) | 1.17 (1.03, 1.31) | 1.17 (1.03, 1.31) | 1.17 (1.03, 1.31) | 1.17 (1.03, 1.31) |
| Diabetes | 1.32 (1.04, 1.60) | 1.31 (1.04, 1.59) | 1.24 (1.03, 1.43) | 1.19 (1.02, 1.35) | 1.15 (1.02, 1.27) | 1.09 (1.01, 1.15) |
| **Processed Meat (per 50g)** | | | | | | |
| Colorectal Cancer | 1.18 (1.09, 1.27) | 1.18 (1.09, 1.27) | 1.18 (1.09, 1.27) | 1.18 (1.09, 1.27) | 1.18 (1.09, 1.27) | 1.18 (1.09, 1.27) |
| IHD | 2.57 (1.05, 4.70) | 2.12 (1.04, 3.50) | 1.52 (1.02, 2.05) | 1.42 (1.02, 1.83) | 1.35 (1.02, 1.69) | 1.25 (1.01, 1.48) |
| Diabetes | 1.94 (1.39, 2.56) | 1.91 (1.38, 2.51) | 1.65 (1.28, 2.05) | 1.51 (1.23, 1.80) | 1.39 (1.18, 1.61) | 1.22 (1.10, 1.33) |
| **Sugar Sweetened Beverage (per 226.8g)** | | | | | | |
| Esophageal Cancer | 1.008 (1.002, 1.017) | 1.008 (1.002, 1.017) | 1.009 (1.002, 1.018) | 1.01 (1.002, 1.02) | 1.01 (1.002, 1.019) | 1.009 (1.002, 1.018) |
| Thyroid Cancer | 1.005 (1.002, 1.009) | 1.005 (1.002, 1.009) | 1.006 (1.002, 1.011) | 1.006 (1.002, 1.011) | 1.006 (1.002, 1.011) | 1.005 (1.002, 1.01) |
| Liver Cancer | 1.007 (1.002, 1.011) | 1.007 (1.002, 1.011) | 1.007 (1.003, 1.013) | 1.008 (1.003, 1.014) | 1.008 (1.003, 1.013) | 1.007 (1.003, 1.012) |
| Pancreatic Cancer | 1.002 (1, 1.004) | 1.002 (1, 1.004) | 1.002 (1, 1.004) | 1.002 (1, 1.005) | 1.002 (1, 1.004) | 1.002 (1, 1.004) |
| Colorectal Cancer | 1.004 (1.003, 1.006) | 1.004 (1.003, 1.006) | 1.005 (1.003, 1.007) | 1.005 (1.003, 1.007) | 1.005 (1.003, 1.007) | 1.005 (1.003, 1.006) |
| Kidney Cancer | 1.006 (1.004, 1.009) | 1.006 (1.004, 1.009) | 1.006 (1.004, 1.009) | 1.007 (1.004, 1.01) | 1.007 (1.004, 1.01) | 1.006 (1.004, 1.009) |
| Leukemia | 1.002 (1.001, 1.003) | 1.002 (1.001, 1.003) | 1.002 (1.001, 1.004) | 1.003 (1.001, 1.004) | 1.002 (1.001, 1.004) | 1.002 (1.001, 1.004) |
| IHD | 1.021 (1.006, 1.039) | 1.019 (1.007, 1.036) | 1.013 (1.009, 1.019) | 1.011 (1.007, 1.015) | 1.008 (1.005, 1.012) | 1.004 (1.002, 1.007) |
| Ischemic Stroke | 1.023 (1.008, 1.041) | 1.022 (1.01, 1.038) | 1.015 (1.01, 1.023) | 1.012 (1.008, 1.018) | 1.008 (1.005, 1.012) | 1.002 (1, 1.004) |
| Hemorrhagic Stroke | 1.028 (1.013, 1.048) | 1.028 (1.013, 1.048) | 1.022 (1.013, 1.033) | 1.016 (1.01, 1.024) | 1.01 (1.006, 1.016) | 1.002 (1, 1.006) |
| Diabetes | 1.46 (1.22, 1.75) | 1.43 (1.18, 1.70) | 1.30 (1.14, 1.48) | 1.24 (1.12, 1.38) | 1.19 (1.09, 1.29) | 1.12 (1.10, 1.15) |
| Chronic Kidney Disease | 1.015 (1.001, 1.032) | 1.015 (1.001, 1.032) | 1.015 (1.001, 1.032) | 1.017 (1.001, 1.035) | 1.014 (1.003, 1.027) | 1.009 (1, 1.025) |

**Note:** All dose-response relative risks were extracted from the 2015 GBD with the exception of sugar sweetened beverages which were extracted from the 2013 GBD
