# Supplementary material for: The economic burden of not meeting food recommendations in Canada: The cost of doing nothing
Source: PLoS One. 2018 Apr 27;13(4):e0196333. doi: 10.1371/journal.pone.0196333 (PMC5922758; doi:10.1371/journal.pone.0196333)
Supplement: S2 Table — (DOCX) [file pone.0196333.s002.docx]

**Supporting Information 2: Percentages of the 2004 Canadian population ≥2 years by age and sex, and by level of food intake**

**Nuts and Seeds Servings (svgs)**

Females

|  | **<0.5 svgs/d** | **0.5-<1 svgs/d** | **≥1 svg/d** |
| --- | --- | --- | --- |
| **≤14 years** | 98.3 | 0.66 | 1.1 |
| **15-34 years** | 96.4 | 2.1 | 1.5 |
| **35-54 years** | 93.9 | 3.0 | 3.1 |
| **55-64 years** | 91.5 | 4.2 | 4.3 |
| **65-74 years** | 95.6 | 2.5 | 1.9 |
| **≥75 years** | 96.8 | 1.7 | 1.5 |

Males

|  | **<0.5 svgs/d** | **0.5-<1 svgs/d** | **≥1 svg/d** |
| --- | --- | --- | --- |
| **≤14 years** | 98.6 | 0.62 | 0.80 |
| **15-34 years** | 95.8 | 1.7 | 2.5 |
| **35-54 years** | 94.8 | 1.8 | 3.5 |
| **55-64 years** | 94.9 | 1.6 | 3.5 |
| **65-74 years** | 93.1 | 4.0 | 3.0 |
| **≥75 years** | 95.5 | 1.7 | 2.8 |

**Whole Grain Servings (svgs)**

Females

|  | **<0.5 svgs/d** | **0.5-<1 svgs/d** | **1-<1.5 svgs/d** | **1.5-<2 svgs/d** | **≥2 svgs/d** |
| --- | --- | --- | --- | --- | --- |
| **≤14 years** | 49.7 | 25.3 | 13.6 | 6.6 | 4.8 |

|  | **<0.5 svgs/d** | **0.5-<1 svgs/d** | **1-<1.5 svgs/d** | **1.5-<2 svgs/d** | **2-<2.5 svgs/d** | **2.5-<3 svgs/d** | **≥3 svgs/d** |
| --- | --- | --- | --- | --- | --- | --- | --- |
| **15-34 years** | 53.5 | 19.2 | 11.9 | 7.4 | 4.2 | 2.2 | 1.6 |
| **35-54 years** | 47.5 | 20.9 | 13.9 | 9.0 | 4.9 | 2.3 | 1.6 |
| **55-64 years** | 36.1 | 22.1 | 17.2 | 11.8 | 6.9 | 3.4 | 2.4 |
| **65-74 years** | 36.6 | 24.1 | 18.2 | 11.2 | 5.8 | 2.5 | 1.5 |
| **≥75 years** | 27.7 | 23.8 | 20.7 | 14.3 | 7.9 | 3.5 | 2.2 |

Males

|  | **<0.5 svgs/d** | **0.5-<1 svgs/d** | **1-<1.5 svgs/d** | **1.5-<2 svgs/d** | **≥2 svgs/d** |
| --- | --- | --- | --- | --- | --- |
| **≤14 years** | 41.1 | 25.0 | 15.6 | 9.2 | 9.1 |

|  | **<0.5 svgs/d** | **0.5-<1 svgs/d** | **1-<1.5 svgs/d** | **1.5-<2 svgs/d** | **2-<2.5 svgs/d** | **2.5-<3 svgs/d** | **3-<3.5 svgs/d** | **3.5-<4 svgs/d** | **≥4 svgs/d** |
| --- | --- | --- | --- | --- | --- | --- | --- | --- | --- |
| **15-34 years** | 57.2 | 16.0 | 9.6 | 6.4 | 4.1 | 2.7 | 1.7 | 0.99 | 1.3 |
| **35-54 years** | 47.1 | 18.1 | 11.6 | 8.3 | 5.7 | 3.7 | 2.3 | 1.4 | 1.8 |

|  | **<0.5 svgs/d** | **0.5-<1 svgs/d** | **1-<1.5 svgs/d** | **1.5-<2 svgs/d** | **2-<2.5 svgs/d** | **2.5-<3 svgs/d** | **3-<3.5 svgs/d** | **≥3.5 svgs/d** |
| --- | --- | --- | --- | --- | --- | --- | --- | --- |
| **55-64 years** | 40.3 | 18.8 | 13.4 | 9.7 | 6.8 | 4.5 | 2.8 | 3.5 |
| **65-74 years** | 34.4 | 19.9 | 15.8 | 11.6 | 7.5 | 4.7 | 2.8 | 3.1 |
| **≥75 years** | 32.5 | 20.9 | 16.8 | 12.2 | 7.6 | 4.6 | 2.5 | 2.8 |

**Fruit Servings (svgs)**

Females

|  | **<0.5 svgs/d** | **0.5-<1 svgs/d** | **1-<1.5 svgs/d** | **1.5-<2 svgs/d** | **≥2 svgs/d** |
| --- | --- | --- | --- | --- | --- |
| **≤14 years** | 16.0 | 24.6 | 23.5 | 16.9 | 19.0 |

|  | **<0.5 svgs/d** | **0.5-<1 svgs/d** | **1-<1.5 svgs/d** | **1.5-<2 svgs/d** | **2-<2.5 svgs/d** | **2.5-<3 svgs/d** | **≥3 svgs/d** |
| --- | --- | --- | --- | --- | --- | --- | --- |
| **15-34 years** | 34.6 | 19.8 | 15.6 | 11.5 | 7.9 | 4.8 | 5.8 |
| **35-54 years** | 25.1 | 18.9 | 16.8 | 13.8 | 10.1 | 6.7 | 8.7 |
| **55-64 years** | 19.9 | 18.9 | 18.4 | 15.6 | 11.1 | 7.2 | 8.9 |
| **65-74 years** | 16.8 | 18.8 | 19.5 | 16.8 | 11.8 | 7.3 | 9.0 |
| **≥75 years** | 15.4 | 19.5 | 20.7 | 17.4 | 11.7 | 7.2 | 8.1 |

Males

|  | **<0.5 svgs/d** | **0.5-<1 svgs/d** | **1-<1.5 svgs/d** | **1.5-<2 svgs/d** | **≥2 svgs/d** |
| --- | --- | --- | --- | --- | --- |
| **≤14 years** | 22.3 | 26.1 | 21.5 | 14.4 | 15.7 |

|  | **<0.5 svgs/d** | **0.5-<1 svgs/d** | **1-<1.5 svgs/d** | **1.5-<2 svgs/d** | **2-<2.5 svgs/d** | **2.5-<3 svgs/d** | **≥3 svgs/d** |
| --- | --- | --- | --- | --- | --- | --- | --- |
| **15-34 years** | 44.1 | 20.9 | 13.0 | 8.3 | 5.2 | 3.2 | 5.2 |
| **35-54 years** | 29.7 | 20.9 | 15.5 | 11.0 | 7.7 | 5.3 | 9.9 |
| **55-64 years** | 22.3 | 20.5 | 16.6 | 12.5 | 9.2 | 6.3 | 12.6 |
| **65-74 years** | 20.1 | 20.5 | 18.1 | 13.6 | 9.6 | 6.4 | 11.6 |
| **≥75 years** | 20.6 | 22.6 | 18.8 | 13.6 | 9.2 | 5.8 | 9.4 |

**Vegetable Servings (svgs)**

Females

|  | **<0.5 svgs/d** | **0.5-<1 svgs/d** | **1-<1.5 svgs/d** | **1.5-<2 svgs/d** | **2-<2.5 svgs/d** | **2.5-<3 svgs/d** | **≥3 svgs/d** |
| --- | --- | --- | --- | --- | --- | --- | --- |
| **≤14 years** | 9.7 | 22.0 | 20.1 | 14.9 | 10.3 | 7.1 | 15.9 |

|  | **<0.5 svgs/d** | **0.5-<1 svgs/d** | **1-<1.5 svgs/d** | **1.5-<2 svgs/d** | **2-<2.5 svgs/d** | **2.5-<3 svgs/d** | **3-<3.5 svgs/d** | **3.5-<4 svgs/d** | **≥4 svgs/d** |
| --- | --- | --- | --- | --- | --- | --- | --- | --- | --- |
| **15-34 years** | 1.4 | 11.7 | 20.4 | 21.1 | 16.5 | 11.4 | 7.2 | 4.4 | 5.8 |
| **35-54 years** | 0.38 | 5.3 | 13.2 | 17.6 | 17.1 | 14.3 | 11.2 | 7.6 | 13.4 |
| **55-64 years** | 0.28 | 4.4 | 11.8 | 16.5 | 16.8 | 14.7 | 11.5 | 8.2 | 15.7 |
| **65-74 years** | 0.39 | 5.1 | 13.4 | 17.7 | 17.5 | 14.4 | 10.6 | 7.6 | 13.1 |
| **≥75 years** | 1.2 | 10.6 | 19.4 | 20.7 | 17.0 | 11.8 | 8.0 | 4.7 | 6.7 |

Males

|  | **<0.5 svgs/d** | **0.5-<1 svgs/d** | **1-<1.5 svgs/d** | **1.5-<2 svgs/d** | **2-<2.5 svgs/d** | **2.5-<3 svgs/d** | **≥3 svgs/d** |
| --- | --- | --- | --- | --- | --- | --- | --- |
| **≤14 years** | 12.9 | 24.8 | 20.3 | 14.2 | 9.4 | 6.2 | 12.2 |

|  | **<0.5 svgs/d** | **0.5-<1 svgs/d** | **1-<1.5 svgs/d** | **1.5-<2 svgs/d** | **2-<2.5 svgs/d** | **2.5-<3 svgs/d** | **3-<3.5 svgs/d** | **3.5-<4 svgs/d** | **4-<4.5 svgs/d** | **4.5-<5 svgs/d** | **≥5 svgs/d** |
| --- | --- | --- | --- | --- | --- | --- | --- | --- | --- | --- | --- |
| **15-34 years** | 3.5 | 13.1 | 16.9 | 15.9 | 13.5 | 10.1 | 7.6 | 5.5 | 4.0 | 2.9 | 7.0 |
| **35-54 years** | 2.3 | 10.4 | 15.0 | 15.2 | 13.4 | 10.8 | 8.7 | 6.4 | 4.8 | 3.5 | 9.4 |

|  | **<0.5 svgs/d** | **0.5-<1 svgs/d** | **1-<1.5 svgs/d** | **1.5-<2 svgs/d** | **2-<2.5 svgs/d** | **2.5-<3 svgs/d** | **3-<3.5 svgs/d** | **3.5-<4 svgs/d** | **≥4 svgs/d** |
| --- | --- | --- | --- | --- | --- | --- | --- | --- | --- |
| **55-64 years** | 1.2 | 6.7 | 11.2 | 13.1 | 13.1 | 11.4 | 9.6 | 7.8 | 26.1 |
| **65-74 years** | 1.6 | 8.4 | 13.3 | 14.4 | 13.6 | 11.3 | 9.0 | 7.2 | 21.3 |
| **≥75 years** | 2.3 | 10.8 | 15.1 | 15.4 | 13.4 | 10.9 | 8.5 | 6.2 | 17.4 |

**Milk Servings (svgs)**

Females

|  | **<0.5 svgs/d** | **0.5-<1 svgs/d** | **1-<1.5 svgs/d** | **1.5-<2 svgs/d** | **≥2 svgs/d** |
| --- | --- | --- | --- | --- | --- |
| **≤14 years** | 22.1 | 22.7 | 16.4 | 11.2 | 27.6 |
| **15-34 years** | 40.3 | 25.9 | 15.6 | 8.5 | 9.7 |
| **35-54 years** | 46.9 | 26.8 | 13.7 | 6.6 | 6.0 |
| **55-64 years** | 52.1 | 25.8 | 11.9 | 5.5 | 4.7 |
| **65-74 years** | 46.2 | 28.4 | 13.5 | 6.4 | 5.5 |
| **≥75 years** | 40.4 | 30.0 | 15.3 | 7.4 | 6.9 |

Males

|  | **<0.5 svgs/d** | **0.5-<1 svgs/d** | **1-<1.5 svgs/d** | **1.5-<2 svgs/d** | **≥2 svgs/d** |
| --- | --- | --- | --- | --- | --- |
| **≤14 years** | 14.8 | 19.2 | 15.9 | 12.3 | 37.8 |
| **15-34 years** | 35.8 | 22.4 | 15.3 | 9.8 | 16.8 |
| **35-54 years** | 50.8 | 24.0 | 12.1 | 6.2 | 6.7 |
| **55-64 years** | 52.8 | 24.0 | 11.6 | 5.8 | 5.8 |
| **65-74 years** | 46.3 | 26.3 | 13.3 | 7.0 | 7.1 |
| **≥75 years** | 34.8 | 27.2 | 16.7 | 9.5 | 11.8 |

**Processed Meat (svgs)**

Females

|  | **<0.25 svgs/d** | **0.25-<0.5 svgs/d** | **0.5-<1 svgs/d** | **1-<1.5 svgs/d** | **1.5-<2 svgs/d** | **≥2 svgs/d** |
| --- | --- | --- | --- | --- | --- | --- |
| **≤14 years** | 49.3 | 36.2 | 13.6 | 0.82 | 0.042 | 0.001 |

|  | **<0.25 svgs/d** | **0.25-<0.5 svgs/d** | **0.5-<1 svgs/d** | **≥1 svgs/d** |
| --- | --- | --- | --- | --- |
| **15-34 years** | 67.0 | 29.9 | 3.1 | 0.026 |
| **35-54 years** | 70.6 | 26.9 | 2.5 | 0.022 |
| **55-64 years** | 78.4 | 20.1 | 1.4 | 0.013 |
| **65-74 years** | 73.2 | 24.6 | 2.2 | 0.016 |

|  | **<0.25 svgs/d** | **0.25-<0.5 svgs/d** | **0.5-<1 svgs/d** | **1-<1.5 svgs/d** | **≥1.5 svgs/d** |
| --- | --- | --- | --- | --- | --- |
| **≥75 years** | 84.0 | 15.2 | 0.85 | 0.007 | 0.001 |

Males

|  | **<0.25 svgs/d** | **0.25-<0.5 svgs/d** | **0.5-<1 svgs/d** | **1-<1.5 svgs/d** | **1.5-<2 svgs/d** | **2-<2.5 svgs/d** | **≥2.5 svgs/d** |
| --- | --- | --- | --- | --- | --- | --- | --- |
| **≤14 years** | 34.4 | 40.3 | 23.0 | 2.2 | 0.15 | 0.013 | 0.001 |

|  | **<0.25 svgs/d** | **0.25-<0.5 svgs/d** | **0.5-<1 svgs/d** | **1-<1.5 svgs/d** | **1.5-<2 svgs/d** | **≥2 svgs/d** |
| --- | --- | --- | --- | --- | --- | --- |
| **15-34 years** | 22.4 | 47.2 | 28.5 | 1.8 | 0.059 | 0.002 |

|  | **<0.25 svgs/d** | **0.25-<0.5 svgs/d** | **0.5-<1 svgs/d** | **1-<1.5 svgs/d** | **≥1.5 svgs/d** |
| --- | --- | --- | --- | --- | --- |
| **35-54 years** | 28.3 | 47.7 | 22.8 | 1.1 | 0.047 |
| **55-64 years** | 26.2 | 47.1 | 25.1 | 1.5 | 0.049 |
| **65-74 years** | 38.1 | 45.5 | 15.7 | 0.57 | 0.013 |
| **≥75 years** | 51.2 | 40.2 | 8.6 | 0.12 | 0.001 |

**Sugar Sweetened Beverages (svgs)**

Females

|  | **<0.25 svgs/d** | **0.25<1 svgs/d** | **1-<1.5 svgs/d** | **1.5-<2 svgs/d** | **2-<3 svgs/d** | **3-<4 svgs/d** | **4-<5 svgs/d** | **5-<6 svgs/d** | **6-<7 svgs/d** | **7-<8 svgs/d** | **≥8 svgs/d** |
| --- | --- | --- | --- | --- | --- | --- | --- | --- | --- | --- | --- |
| **≤14 years** | 15.8 | 39.1 | 17.3 | 11.3 | 11.2 | 3.6 | 1.1 | 0.34 | 0.10 | 0.030 | 0.013 |

|  | **<0.25 svgs/d** | **0.25<1 svgs/d** | **1-<1.5 svgs/d** | **1.5-<2 svgs/d** | **2-<3 svgs/d** | **3-<4 svgs/d** | **4-<5 svgs/d** | **5-<6 svgs/d** | **6-<7 svgs/d** | **7-<8 svgs/d** | **8-<9 svgs/d** | **9-<10 svgs/d** | **≥10 svgs/d** |
| --- | --- | --- | --- | --- | --- | --- | --- | --- | --- | --- | --- | --- | --- |
| **15-34 years** | 24.6 | 30.1 | 13.0 | 10.0 | 12.8 | 5.9 | 2.4 | 0.88 | 0.30 | 0.11 | 0.036 | 0.016 | 0.007 |

|  | **<0.25 svgs/d** | **0.25<1 svgs/d** | **1-<1.5 svgs/d** | **1.5-<2 svgs/d** | **2-<3 svgs/d** | **3-<4 svgs/d** | **4-<5 svgs/d** | **5-<6 svgs/d** | **6-<7 svgs/d** | **≥7 svgs/d** |
| --- | --- | --- | --- | --- | --- | --- | --- | --- | --- | --- |
| **35-54 years** | 48.7 | 29.9 | 8.8 | 5.4 | 5.0 | 1.6 | 0.46 | 0.14 | 0.031 | 0.013 |

|  | **<0.25 svgs/d** | **0.25<1 svgs/d** | **1-<1.5 svgs/d** | **1.5-<2 svgs/d** | **2-<3 svgs/d** | **3-<4 svgs/d** | **4-<5 svgs/d** | **≥5 svgs/d** |
| --- | --- | --- | --- | --- | --- | --- | --- | --- |
| **55-64 years** | 57.7 | 27.1 | 6.9 | 3.8 | 3.3 | 0.90 | 0.25 | 0.090 |

|  | **<0.25 svgs/d** | **0.25<1 svgs/d** | **1-<1.5 svgs/d** | **1.5-<2 svgs/d** | **2-<3 svgs/d** | **3-<4 svgs/d** | **≥4 svgs/d** |
| --- | --- | --- | --- | --- | --- | --- | --- |
| **65-74 years** | 61.7 | 26.1 | 5.9 | 3.2 | 2.4 | 0.55 | 0.17 |

|  | **<0.25 svgs/d** | **0.25<1 svgs/d** | **1-<1.5 svgs/d** | **1.5-<2 svgs/d** | **2-<3 svgs/d** | **≥3 svgs/d** |
| --- | --- | --- | --- | --- | --- | --- |
| **≥75 years** | 66.7 | 24.5 | 4.9 | 2.2 | 1.5 | 0.28 |

Males

|  | **<0.25 svgs/d** | **0.25<1 svgs/d** | **1-<1.5 svgs/d** | **1.5-<2 svgs/d** | **2-<3 svgs/d** | **3-<4 svgs/d** | **4-<5 svgs/d** | **5-<6 svgs/d** | **6-<7 svgs/d** | **7-<8 svgs/d** | **8-<9 svgs/d** | **9-<10 svgs/d** | **≥10 svgs/d** |
| --- | --- | --- | --- | --- | --- | --- | --- | --- | --- | --- | --- | --- | --- |
| **≤14 years** | 12.5 | 35.2 | 17.4 | 12.4 | 14.1 | 5.5 | 2.0 | 0.64 | 0.22 | 0.074 | 0.021 | 0.012 | 0.005 |

|  | **<0.25 svgs/d** | **0.25<1 svgs/d** | **1-<1.5 svgs /d** | **1.5-<2 svgs/d** | **2-<3 svgs/d** | **3-<4 svgs/d** | **4-<5 svgs /d** | **5-<6 svgs /d** | **6-<7 svgs /d** | **7-<8 svgs /d** | **8-<9 svgs /d** | **9-<10 svgs /d** | **10-<11 svgs /d** | **11-<12 svgs /d** | **12-<13 svgs /d** | **13-<14 svgs /d** | **≥14 svgs /d** |
| --- | --- | --- | --- | --- | --- | --- | --- | --- | --- | --- | --- | --- | --- | --- | --- | --- | --- |
| **15-34 years** | 9.9 | 22.8 | 12.6 | 11.0 | 17.8 | 12.0 | 7.0 | 3.6 | 1.8 | 0.85 | 0.36 | 0.16 | 0.071 | 0.023 | 0.014 | 0.005 | 0.005 |

|  | **<0.25 svgs/d** | **0.25<1 svgs/d** | **1-<1.5 svgs/d** | **1.5-<2 svgs/d** | **2-<3 svgs/d** | **3-<4 svgs/d** | **4-<5 svgs/d** | **5-<6 svgs/d** | **6-<7 svgs/d** | **7-<8 svgs/d** | **8-<9 svgs/d** | **9-<10 svgs/d** | **≥10 svgs/d** |
| --- | --- | --- | --- | --- | --- | --- | --- | --- | --- | --- | --- | --- | --- |
| **35-54 years** | 29.3 | 32.5 | 11.7 | 8.0 | 10.0 | 5.0 | 2.1 | 0.88 | 0.34 | 0.12 | 0.045 | 0.017 | 0.023 |

|  | **<0.25 svgs/d** | **0.25<1 svgs/d** | **1-<1.5 svgs/d** | **1.5-<2 svgs/d** | **2-<3 svgs/d** | **3-<4 svgs/d** | **4-<5 svgs/d** | **5-<6 svgs/d** | **≥6 svgs/d** |
| --- | --- | --- | --- | --- | --- | --- | --- | --- | --- |
| **55-64 years** | 42.5 | 32.4 | 9.6 | 5.9 | 6.2 | 2.4 | 0.74 | 0.25 | 0.11 |

|  | **<0.25 svgs/d** | **0.25<1 svgs/d** | **1-<1.5 svgs/d** | **1.5-<2 svgs/d** | **2-<3 svgs/d** | **3-<4 svgs/d** | **4-<5 svgs/d** | **≥5 svgs/d** |
| --- | --- | --- | --- | --- | --- | --- | --- | --- |
| **65-74 years** | 54.4 | 29.5 | 7.2 | 3.9 | 3.6 | 1.1 | 0.32 | 0.12 |

|  | **<0.25 svgs/d** | **0.25<1 svgs/d** | **1-<1.5 svgs/d** | **1.5-<2 svgs/d** | **2-<3 svgs/d** | **3-<4 svgs/d** | **≥4 svgs/d** |
| --- | --- | --- | --- | --- | --- | --- | --- |
| **≥75 years** | 68.9 | 23.6 | 4.3 | 1.9 | 1.1 | 0.20 | 0.040 |

**Red Meat Servings (svgs)**

Females

|  | **<0.5 svgs/d** | **0.5-<1 svgs/d** | **1-<1.5 svgs/d** | **≥1.5 svgs/d** |
| --- | --- | --- | --- | --- |
| **≤14 years** | 70.1 | 29.6 | 0.38 | 0.001 |
| **15-34 years** | 59.2 | 38.9 | 1.8 | 0.007 |
| **35-54 years** | 44.2 | 50.8 | 4.9 | 0.075 |
| **55-64 years** | 42.0 | 51.3 | 6.6 | 0.14 |
| **65-74 years** | 48.0 | 48.2 | 3.8 | 0.035 |
| **≥75 years** | 49.8 | 47.0 | 3.1 | 0.025 |

Males

|  | **<0.5 svgs/d** | **0.5-<1 svgs/d** | **1-<1.5 svgs/d** | **≥1.5 svgs/d** |
| --- | --- | --- | --- | --- |
| **≤14 years** | 54.8 | 43.3 | 1.9 | 0.015 |

|  | **<0.5 svgs/d** | **0.5-<1 svgs/d** | **1-<1.5 svgs/d** | **1.5-<2 svgs/d** | **2-<2.5 svgs/d** | **2.5-<3 svgs/d** | **3-<3.5 svgs/d** | **3.5-<4 svgs/d** | **4-<4.5 svgs/d** | **4.5-<5 svgs/d** | **5-<5.5 svgs/d** | **≥5.5 svgs/d** |
| --- | --- | --- | --- | --- | --- | --- | --- | --- | --- | --- | --- | --- |
| **15-34 years** | 22.4 | 37.1 | 23.6 | 10.9 | 4.0 | 1.4 | 0.43 | 0.15 | 0.032 | 0.005 | 0.001 | 0.001 |
| **35-54 years** | 17.1 | 35.3 | 25.7 | 13.3 | 5.6 | 2.0 | 0.69 | 0.24 | 0.064 | 0.019 | 0.005 | 0.001 |

|  | **<0.5 svgs/d** | **0.5-<1 svgs/d** | **1-<1.5 svgs/d** | **1.5-<2 svgs/d** | **2-<2.5 svgs/d** | **2.5-<3 svgs/d** | **3-<3.5 svgs/d** | **3.5-<4 svgs/d** | **4-<4.5 svgs/d** | **4.5-<5 svgs/d** | **≥5 svgs/d** |
| --- | --- | --- | --- | --- | --- | --- | --- | --- | --- | --- | --- |
| **55-64 years** | 22.1 | 36.7 | 23.4 | 11.0 | 4.5 | 1.6 | 0.45 | 0.17 | 0.048 | 0.006 | 0.007 |

|  | **<0.5 svgs/d** | **0.5-<1 svgs/d** | **1-<1.5 svgs/d** | **1.5-<2 svgs/d** | **2-<2.5 svgs/d** | **2.5-<3 svgs/d** | **3-<3.5 svgs/d** | **3.5-<4 svgs/d** | **≥4 svgs/d** |
| --- | --- | --- | --- | --- | --- | --- | --- | --- | --- |
| **65-74 years** | 27.9 | 38.7 | 20.9 | 8.6 | 2.8 | 0.83 | 0.24 | 0.057 | 0.019 |

|  | **<0.5 svgs/d** | **0.5-<1 svgs/d** | **1-<1.5 svgs/d** | **1.5-<2 svgs/d** | **2-<2.5 svgs/d** | **2.5-<3 svgs/d** | **3-<3.5 svgs/d** | **≥3.5 svgs/d** |
| --- | --- | --- | --- | --- | --- | --- | --- | --- |
| **≥75 years** | 36.3 | 38.8 | 17.1 | 5.6 | 1.7 | 0.44 | 0.12 | 0.022 |
